# Anxiety and depression associated with a positive prostate biopsy result: A comparative, prospective cohort study

**DOI:** 10.1590/S1677-5538.IBJU.2019.0719

**Published:** 2020-09-02

**Authors:** Ertugrul Sefik, Bulent Gunlusoy, Anil Eker, Serdar Celik, Yasin Ceylan, Asli Koskderelioglu, Ismail Basmaci, Tansu Degirmenci

**Affiliations:** 1 Department of Urology Health Sciences University Bozyaka Training and Research Hospital Izmir Turkey Department of Urology, Health Sciences University, Bozyaka Training and Research Hospital, Izmir, Turkey;; 2 Department of Neurology Health Sciences University Bozyaka Training and Research Hospital Izmir Turkey Department of Neurology, Health Sciences University, Bozyaka Training and Research Hospital, Izmir, Turkey

**Keywords:** Prostate-Specific Antigen, Biopsy, Anxiety, Depression

## Abstract

**Purpose:**

To investigate the course of anxiety and depression before and after transrectal ultrasound-guided prostate biopsy (TRUS-Bx) and in the postoperative 1st month when the histopathological biopsy result was obtained.

**Methods:**

In between June 2017- January 2019, 204 patients who underwent TRUS-Bx and completed the questionnaires assessing anxiety and depression were included in the study. Questionnaires were completed immediately before the biopsy, immediately after the biopsy and at the end of the first month when the histopathological biopsy results were given. State-Trait Anxiety Inventory (STAI), Hospital Anxiety and Depression Scale (HADS) and perceived stress scale (PSS) forms were used to assess anxiety and depression. After the histopathological examination patients were divided into two groups as patients without cancer (Group 1) and with cancer (Group 2). Data was compared between the groups.

**Results:**

PSA level was negatively correlated with STAI TX-1 scores of the patients immediately after TRUS-Bx, whereas it was positively correlated with STAI TX-1 and TX-2 30 days after the TRUS-Bx. PSA level was positively correlated with HADS-A and HADS-D scores immediately before and 30 days after TRUS-Bx. Biopsy results showed a significant difference in 30 day post-biopsy related data. STAI TX-1, STAI TX-2, HADS-A, HADS-D and PSS scores were higher in Group 2 compared with Group 1.

**Conclusions:**

Pre-biopsy anxiety disappeared after bx, but there was a significant increase in anxiety and depression in patients after the diagnosis of malignancy. Patients were seriously concerned about the diagnosis of prostate cancer.

## INTRODUCTION

Through the widespread use of prostate-specific antigen (PSA), earlier detection of prostate cancer (PCa) at lower stages, lower grades, and smaller tumor volumes is feasible. Contemporary PCa screening modalities such as PSA and digital rectal examination (DRE) are two parameters used to predict the detection of PCa ( 1 ). Transrectal ultrasound-guided biopsy of the prostate (TRUS-Bx) is considered the standard of care for diagnosis of PCa. For men who have an elevated PSA test, histologic diagnosis of PCa requires TRUS-Bx, and the numbers of men undergoing biopsy have increased correspondingly ( 2 ). Rosario et al. stated that TRUS-Bx is well tolerated by most men, but it is associated with significant symptoms in a minority and affects attitudes to repeat biopsy and primary care resource use ( 3 ).

Prostate biopsy can cause psychological and physical impacts ( 4 ). Cancer-related worry or diagnosis of PCa and evidence of the psychological impact of biopsy can lead to increased anxiety and depression ( 5 - 7 ). The high prevalence of severe distress after PCa diagnosis has resulted in the recommendation that interventions target treatment decision-related distress for all men and in-depth psychological support be offered for those who experience ongoing difficulties ( 8 ).

Wade et al. investigated the psychological impact of TRUS-Bx, including relationships between physical biopsy-related symptoms and anxiety/depression using the Hospital Anxiety and Depression Scale (HADS) in a prospective observational study of 1144 men undergoing standard TRUS-Bx ( 5 ). In another study, Korfage et al. recommended clinicians attempt early detection of patients at risk of high levels of anxiety and depression after PCa diagnosis since prevalence is high ( 9 ). As unrecognized, untreated depression and anxiety have serious consequences, clinicians want depression to be clearly demonstrated to decide which variables might serve as signals to screen for depression and anxiety among this older population ( 10 ). It is not clear whether the anxiety and depression observed before and after TRUS-Bx is due to the procedure or is associated with the diagnosis of prostate cancer.

In this prospective study, we aimed to investigate the course of anxiety and depression before and after TRUS-Bx and to evaluate the factors that affect patient anxiety and depression.

## MATERIALS AND METHODS

After receiving ethical approval from the ethics committee of our institution (approval no: 2017/1) and the written informed consent form from all participants, patients with the ability to read and answer the questionnaire forms who underwent prostate biopsy due to elevated PSA or abnormal digital rectal examination from June 2017 to January 2019 were prospectively evaluated. Patients were excluded if they were under 40 years old, had prior prostate biopsy, had a diagnosed psychiatric disease such as psychosis, depression, mental retardation, and dementia, prior or concomitant malignancies, history of serious cardiovascular disease and end stage renal disease.

Patient’s age, PSA level, education level, rectal examination result, prostate volume, pain scores (rated according to the Visual Analogue Scale (VAS) just after the biopsy procedure) and TRUS-Bx pathological results were recorded. Patients underwent standardized 12-core TRUS-Bx with periprostatic blockage. Patients received antibiotic therapy for 5 days from the day of biopsy.

All patients who participated in the study completed the self-report questionnaires assessing anxiety and depression immediately before biopsy, immediately after biopsy and 30 days after biopsy when the histopathological biopsy results were given. In all groups, correlation between patient’s demographics and questionnaire results was investigated. After the histopathological biopsy results were given, patients were divided into two groups as benign pathology of the prostate (Group 1) and PCa (Group 2). Then, patient’s demographics and questionnaire results were compared between the groups. In addition, ∆ scores for the questionnaires, which is the difference between immediately before biopsy and 30 days after biopsy, were compared between the groups. Patients with clinically insignificant PCa (defined as grade group (GG) 1 PCa detected in ≤2 cores and <50% of any one core) and clinically significant PCa (others) were recorded. Correlation between patient’s demographics and questionnaire results was also investigated in group 2.

### Questionnaires

Anxiety and depression were measured using the State-Trait Anxiety Inventory (STAI), Hospital Anxiety and Depression Scale (HADS) (Appendix) and perceived stress scale (PSS) forms. The STAI form includes two scales: State anxiety scale (TX-1) (20 questions, scored between 20 to 80 points) and Trait anxiety scale (TX-2) (20 questions, scored between 20 to 80 points) ( 11 ). The scores on this form were calculated by considering the inverse statements. The Hospital Anxiety and Depression Scale (HADS) also includes two scales: in the questionnaire the 7 odd numbered questions from the 14 questions calculate anxiety (HADS-A), while 7 even numbered questions of the 14 questions calculate depression (HADS-D) ( 12 ). The perceived stress scale (PSS) includes 14 questions. The scores on this form were also calculated considering the 7 inverse statements.

### Statistical Analysis

Data were analyzed using the Statistical Package for Social Sciences, version 20.0 (SPSS, Chicago, Ill) software program. The sample size was calculated according to the power analysis (with 80% power and 5% type I error rate) of the previous studies’ results, the minimum number for the sample was found to be 57patients for anxiety and depression scores in prostate cancer and benign groups. Therefore, we included a total of 204 patients in the current study. The power of this study was found to be 0.665 (p=0.018, CI:-2.51/-0.43) using variance analysis. The Pearson Correlation test was used between questionnaire scores and the age, PSA level and education level. The Mann-Whitney U test and Pearson Chi-square test were used for univariate analysis between the groups according to histopathologic examination results. Also, the Pearson Correlation test was used for questionnaire scores and age, PSA level, VAS score, presence of clinically significant PCa and education level in the PCa group. Data are given as mean±SD in the tables. Statistical significance was defined as p <0.05.

## RESULTS

A total of 204 patients who met the inclusion criteria consented to participate and completed the questionnaires. Patient’s demographic data are given in Table-1 . The mean age of all patients was 64.8±6.7 (48-78) years and PSA level was 10.7±14.9 (0.9-128.8) ng/mL. Abnormal rectal examination rate was 35.3% among the patients. Scores of all questionnaires before the procedure, immediately after and 30 days after the TRUS-Bx (postoperative 1^st^ month) are shown in Table-1 . PCa was diagnosed in 62 patients (30%). The most common ISUP grade was Grade 1 (n=32). Correlation results between the questionnaire scores and the age, PSA level, and VAS scores are shown in Table-2 . According to the correlation results, age was negatively correlated with STAI TX-1 scores immediately after TRUS-Bx. PSA level was negatively correlated with STAI TX-1 scores immediately after TRUS-Bx, whereas it was positively correlated with STAI TX-1 and TX-2 in the postoperative 1^st^month. Also, PSA level was positively correlated with HADS-A and HADS-D scores immediately before TRUS-Bx and in postoperative 1^st^month and positive correlation was found between PSS level in thepostoperative 1^st^month and PSA level. In addition, VAS score was positively correlated with HADS-A immediately after TRUS-Bx and postoperative 1st month PSS. Education level was not correlated with any score. Demographic data and questionnaire results for the benign and malignant (PCa) groups according to histopathological examination results after TRUS-Bx are given in Table-3 . In the comparison of the groups, postoperative 1st month and ∆ levels of STAI TX-1, STAI TX-2, HADS-A, HADS-D and PSS scores were higher in Group 2 compared with Group 1 (p <0.001), whereas immediately before TRUS-BX the STAI TX-1, STAI TX-2, HADS-A, HADS-D and PSS scores were similar between the groups. PSA level was positively correlated with HADS-A and HADS-D before TRUS-Bx, whereas it was correlated with HADS-D in the postoperative 1st month in the PCa group.

**Table 1:** Patients’ demographics and questionnaire results.

Variable		n=204
Age (years), mean ± SD (min-max)		64.8±6.7 (48-78)
PSA (ng/mL), mean ± SD (min-maks)		10.7±14.9 (0.9-128.8)
Prostate volume (cc), mean ± SD (min-max)		55.9±25.6 (20-135)
	Normal	132 (64.7)
DRE , n (%)	Abnormal	72 (35.3)
**Education , n (%)**	illiterate	16 (7.8)
	Primary school	128 (62.7)
	High school	48 (23.5)
	Graduate	12 (5.9)
Before TRUS-BX, STAI, mean ± SD (min-max)	TX-1	38.7±10.4 (20-64)
	TX-2	42.2±8 (21-64)
Before TRUS-BX, HADS, mean ± SD (min-max)	HADS-A	5.5±3.2 (0-14)
	HADS-D	5.7±3.4 (0-16)
**Before TRUS-BX, PSS-14, mean ± SD (min-max)**		**22.1±6.3 (2-33)**
After TRUS-BX, STAI, mean ± SD (min-max)	TX-1	38.1±9.8 (20-57)
	TX-2	40.1±6.7 (24-56)
After TRUS-BX, HADS, mean ± SD (min-max)	HADS-A	5.2±3.4 (0-14)
	HADS-D	5.6±3.5 (0-15)
**After TRUS-BX, PSS-14, mean ± SD (min-max)**		20.7-6.4 (0-34)
Postoperative 1 st month STAI, mean ± SD (min-max)	TX-1	31.5±12.1 (20-59)
	TX-2	32.8±12.3 (20-60)
Postoperative 1 st month HADS, mean ± SD (min-max)	HADS-A	3.6±3.4 (0-16)
	HADS-D	3.2±3.2 (0-13)
Postoperative 1st month PSS-14, mean ± SD (min-max)		17.7±7.9 (5-34)

**PSA =** Prostate specific antigen; **TRUSG-Bx =** Transrectal ultrasound-guided prostate biopsy; **DRE =** Digital rectal examination; **STAI =** State-Trait Anxiety Inventory; **HADS =** Hospital Anxiety and Depression Scale; **PSS =** Perceived stress scale

**Table 2:** Correlation results between the questionnaire scores and the age and PSA level.

		Age	PSA	VAS score
Before TRUS-BX, STAI	TX-1	R=-0.016, p=0.877	R=0.1, p=0.316	R=0.105, p=0.292
	TX-2	R=-0.047, p=0.640	R=0.156, p=0.117	R=-0.037, p=0.712
Before TRUS-BX, HADS	HADS-A	R=-0.065, p=0.518	**R=0.211, p=0.033**	R=0.103, p=0.304
	HADS-D	R=0.041, p=0.682	**R=0.255, p=0.010**	R=-0.066, p=0.511
Before TRUS-BX PSS-14		R=-0.032, p=0.749	R=0.131, p=0.19	R=0.112, p=0.263
After TRUS-BX STAI	TX-1	**R=-0.224, p=0.024**	**R=-0.224, p=0.024**	R=0.117, p=0.241
	TX-2	R=-0.132, p=0.184	R=-0.132, p=0.184	R=0.162, p=0.104
After TRUS-BX, HADS	HADS-A	R=-0.181, p=0.068	R=-0.181, p=0.068	**R=0.280, p=0.004**
	HADS-D	R=-0.121, p=0.225	R=-0.121, p=0.225	R=0.159, p=0.111
After TRUS-BX PSS-14		R=-0.116, p=0.249	R=-0.116, p=0.249	R=0.120, p=0.231
Postoperative 1^st^ month STAI	TX-1	R=0.042, p=0.674	**R=0.256, p=0.009**	R=0.117, p=0.243
	TX-2	R=0.067, p=0.503	**R=0.260, p=0.008**	R=0.060, p=0.547
Postoperative 1 ^st^ month HADS	HADS-A	R=-0.115, p=0.250	**R=0.294, p=0.003**	R=0.189, p=0.057
	HADS-D	R=0.022, p=0.225	**R=0.402, P<0.001**	R=0.074, p=0.457
Postoperative 1 ^st^ month PSS-14		R=0.054, p=0.593	**R=0.286, p=0.004**	**R=0.235, p=0.017**

**Table 3:** Demographic data and questionnaire results of the benign and prostate cancer groups according to histopathological examination results after TRUS-Bx.

Variables : median (min-max)		Group 1 (Benign) (n=142)	Group 2 (prostate cancer ) (n=62)	p
Age (years)		64 (48-78)	69 (48-78)	0.135
PSA (ng/mL)		6 (0.9-37)	8 (3.6-128,8)	0.006
Prostat volume (cc)		55 (25-120)	40 (24.3-135)	<0.001
DRE, n (%)	Normal	102 (71.8)	30 (48.4)	0.005
	Abnormal	40 (28.2)	32 (51.6)	
STAI TX-1	Before TRUS-BX	39 (20-64)	41 (23-58)	0.346
	Postoperative 1 ^st^ month	22 (20-59)	46 (20-58)	**<0.001**
	∆ STAI TX-1	-10 (-35 - +19)	6 (-21 - +21)	<0.001
STAI TX-2	Before TRUS-BX	42 (21-64)	43 (31-60)	0.469
	Postoperative 1 ^st^ month	24 (20-59)	49 (34-60)	**<0.001**
	∆ STAI TX-2	-15 (-35 - +5)	4 (-17 - +21)	**<0.001**
HADS-A	Before TRUS-BX	6 (0-14)	6 (1-13)	0.406
	Postoperative 1 ^st^ month	2 (0-16)	7 (1-13)	**<0.001**
	∆ HADS-A	-2 (-9 - +7)	1 (-7 - +6)	**<0.001**
HADS-D	Before TRUS-BX	5 (0-16)	7 (0-13)	0.322
	Postoperative 1 ^st^ month	2 (0-9)	7 (1-13)	**<0.001**
	∆ HADS-D	-3 (-12 - +2)	0 (-5 - +4)	**<0.001**
PSS-14	Before TRUS-BX	22 (2-33)	23 (10-33)	0.281
	Postoperative 1 ^st^ month	12 (5-27)	29 (15-34)	**<0.001**
	∆ PSS-14	-9 (-18 - +8)	4 (-8 - +13)	**<0.001**

There were 52 patients with clinically significant PCa and 10 with clinically insignificant PCa. No significant correlations were found between presence of clinically significant PCa and anxiety and depression scores.

## DISCUSSION

TRUS-Bx is a procedure that can cause significant pain and discomfort in a great proportion of patients ( 7 ). Anxiety and depression may be observed in patients undergoing prostate cancer screening or in those diagnosed with cancer ( 13 ). Being diagnosed with a malignant tumor puts a considerable psychological burden on patients and their families ( 14 ). The risks and benefits of the procedure should be discussed with the patient before TRUS-Bx. This information can also help to decide whether or not to perform a PSA scan ( 15 ). Macefield et al. found that the psychological effect was highest during the procedure and distress and tense/anxious mood were observed in 20% of all patients ( 16 ). Even after a negative biopsy result, 10% of men still experienced high distress up to 3 months following prostate biopsies ( 17 ). On the other hand, prostate biopsy is the corner stone of active surveillance protocols. Repeat TRUS-Bx is recommended after 1, 4 and 7 years in the surveillance protocol in the Prostate Cancer Research International Active Surveillance (PRIAS) study ( 18 ). Steginga et al. stated decision-related distress was more often present in patients who were undecided about what treatment to receive, and final treatment choice does not cause psychological distress associated with Pca ( 8 ). Minervini et al.stated that anxiety was reduced 35 days after TRUS-Bx even in patients experiencing biopsy-related side effects, except for patients diagnosed with cancer ( 6 ). In our prospective study, we aimed to compare the course of anxiety and depression in those diagnosed with and without cancer by using questionnaires before and after biopsy and after histopathological results.

Men with consistently elevated PSA values may experience psychological distress in relation with the interval of testing, PSA changes, and biopsy results ( 19 ). PSA is an organ-specific marker not specific to cancer. PSA is easily affected by non-cancer factors. It is difficult for physicians to decide about performing TRUS-Bx in patients with elevating PSA levels during follow-up ( 19 ). Also, PSA changes may cause anxiety in a small number of patients. Zhang et al. stated that young age (below 60 yr), poor family economic status, and high level of PSA (above 10ng/mL) were all risk factors for anxiety ( 20 ). Some patients with high anxiety may benefit from additional support during and after TRUS-Bx ( 21 ). In the current study, PSA level was positively correlated with HADS-A (R=0.211, p=0.033 and R=0.294, p=0.003) and HADS-D (R=0.255, p=0.010 and R=0.492, p <0.001) scores immediately before TRUS-Bx and 30 days after TRUS-Bx, respectively. On the other hand, HADS-A (R=-0.181, p=0.068) and HADS-D (R=-0.121, p=0.225) were not significant after TRUS-Bx. The pre-biopsy patient’s symptoms were shown to disappear rapidly after biopsy. The biopsy is well tolerated by the patient if the patient is well-informed prior to biopsy. The method of biopsy, the anesthesia technique and pain related to biopsy should be described in detail. This is especially important for younger patients with anxiety continuing after biopsy (STAI TX-1; R=-0.224, p=0.024). Also, positive correlation was found between PSS level 30 days after TRUS-Bx and PSA level. In addition, VAS score was positively correlated with postoperative HADS-A and 1st month PSS.

Anxiety may occur in 30.5% of patients with PCa ( 22 ). Anxiety in patients with PCa includes state and trait components ( 23 ). The multidimensional feature of anxiety in PCa patients necessitates differentiation of normal psychological reaction to cancer (state), anxious personality predisposition (trait) and symptoms of a psychiatric disorder ( 24 ). Anxiety in PCa includes disease anxiety, elevated PSA anxiety, and concern about recurrence. Fowler et al. reported that 25% of patients with a negative TRUS-Bx result had worried “some of the time” or “a lot” about having cancer one year later ( 25 ).

Our study revealed that the patients’ pre-biopsy symptoms disappeared after biopsy, while there was a significant increase in anxiety and depression in patients after the diagnosis of malignancy. We found that our patients were more worried about cancer than the biopsy procedure. Biopsy results caused a significant difference in 30-day post-biopsy related data. STAI TX-1, STAI TX-2, HADS-A, HADS-D and PSS scores were higher in PCa patients compared with those with benign biopsies. We believe that anxiety can be relieved by explaining to the patients that a significant proportion of patients have clinically insignificant cancer diagnosed after cancer screening and the prognosis of these patients is good. In our study group, 10/62 (16.1%) of PCa patients had clinically insignificant PCa and active surveillance was recommended for these patients. Offering an active surveillence protocol or definitive treatment to the patients did not make a significant difference to their anxiety and depression scores.

There are some limitations of the current study. Our analyses stratified by biopsy result were based on small numbers and for that reason, should be considered exploratory. Another limitation is that we could not follow the psychological distress of confirmed biopsy in the patient with active surveillance approximately one year after the first biopsy. We are uncertain about the willingness of the patients to decide to repeat the biopsy and to reply to the same repeated questionnaires. Also, it is better to follow-up about the psychological susceptibility to PSA changes in patients with benign biopsy. Despite its limitations, the study assessed the psychological stress of patients who underwent TRUS-Bx and were diagnosed with PCa using a highly specialized questionnaire.

## CONCLUSIONS

We observed that anxiety and depression before prostate biopsy disappeared in the early post-biopsy period and increased again after the diagnosis of prostate cancer. Diagnosis of PCa causes more anxiety and depression than TRUS-Bx itself.
