# Supplementary material for: Anxiety and depression associated with a positive prostate biopsy result: A comparative, prospective cohort study
Source: Int Braz J Urol. 2020 Sep 2;46(6):993–1005. doi: 10.1590/S1677-5538.IBJU.2019.0719 (PMC7527087; doi:10.1590/S1677-5538.IBJU.2019.0719)
Supplement: Supplementary file 1 [file 1677-6119-ibju-46-06-0993-suppl01.pdf]

APPENDIX

Questionnaire: Hospital Anxiety and Depression Scale (HADS)

Hospital Anxiety and Depression Scale (HADS)

Tick the box beside the reply that is closest to how you have been feeling in the past week.
Don't take too long over you replies: your immediate is best.

D	A		D	A	
		**I feel tense or 'wound up':**			**I feel as if I am slowed down:**
	3	Most of the time	3		Nearly all the time
	2	A lot of the time	2		Very often
	1	From time to time, occasionally	1		Sometimes
	0	Not at all	0		Not at all
		**I still enjoy the things I used to enjoy:**			**I get a sort of frightened feeling like 'butterflies' in the stomach:**
0		Definitely as much		0	Not at all
1		Not quite so much		1	Occasionally
2		Only a little		2	Quite Often
3		Hardly at all		3	Very Often
		**I get a sort of frightened feeling as if something awful is about to happen:**			**I have lost interest in my appearance:**
	3	Very definitely and quite badly	3		Definitely
	2	Yes, but not too badly	2		I don't take as much care as I should
	1	A little, but it doesn't worry me	1		I may not take quite as much care
	0	Not at all	0		I take just as much care as ever
		**I can laugh and see the funny side of things:**			**I feel restless as I have to be on the move:**
0		As much as I always could		3	Very much indeed
1		Not quite so much now		2	Quite a lot
2		Definitely not so much now		1	Not very much
3		Not at all		0	Not at all
		**Worrying thoughts go through my mind:**			**I look forward with enjoyment to things:**
	3	A great deal of the time	0		As much as I ever did
	2	A lot of the time	1		Rather less than I used to
	1	From time to time, but not too often	2		Definitely less than I used to
	0	Only occasionally	3		Hardly at all
		**I feel cheerful:**			**I get sudden feelings of panic:**
3		Not at all		3	Very often indeed
2		Not often		2	Quite often
1		Sometimes		1	Not very often
0		Most of the time		0	Not at all
		**I can sit at ease and feel relaxed:**			**I can enjoy a good book or radio or TV program:**
	0	Definitely	0		Often
	1	Usually	1		Sometimes
	2	Not Often	2		Not often
	3	Not at all	3		Very seldom

Please check you have answered all the questions

Scoring:
Total score: Depression (D) ___________ Anxiety (A) _______________
0-7 = Normal
8-10 = Borderline abnormal (borderline case)
11-21 = Abnormal (case)

Perceived Stress Scale

The questions in this scale ask you about your feelings and thoughts during the last month. **In each case, you will be asked to indicate by circling *how often* you felt or thought a certain way.**

Name ______________________________________ Date ___________

Age ______ Gender (*Circle*): **M F** Other ________________________

0 = Never 1 = Almost Never 2 = Sometimes 3 = Fairly Often 4 = Very Often

1. In the last month, how often have you been upset because of something that happened unexpectedly?	0	1	2	3	4
2. In the last month, how often have you felt that you were unable to control the important things in your life?	0	1	2	3	4
3. In the last month, how often have you felt nervous and "stressed"?	0	1	2	3	4
4. In the last month, how often have you felt confident about your ability to handle your personal problems?	0	1	2	3	4
5. In the last month, how often have you felt that things were going your way?	0	1	2	3	4
6. In the last month, how often have you found that you could not cope with all the things that you had to do?	0	1	2	3	4
7. In the last month, how often have you been able to control irritations in your life?	0	1	2	3	4
8. In the last month, how often have you felt that you were on top of things?	0	1	2	3	4
9. In the last month, how often have you been angered because of things that were outside of your control?	0	1	2	3	4
10. In the last month, how often have you felt difficulties were piling up so high that you could not overcome them?	0	1	2	3	4

SELF-EVALUATION QUESTIONNAIRE STAI Form Y-1

Please provide the following information:

Name____Date_____________S_____

Age__________________ Gender (*Circle*) **M** **F** T_____

DIRECTIONS:

A number of statements which people have used to describe themselves are given below. Read each statement and then circle the appropriate number to the right of the statement to indicate how you feel *right* now, that is, *at this moment*. There are no right or wrong answers. Do not spend too much time on any one statement but give the answer which seems to describe your present feelings best.

	NOT AT ALL	SOMEWHAT	MODERATELY SO	VERY MUCH SO
1. I feel calm	1	2	3	4
2. I feel secure	1	2	3	4
3. I am tense	1	2	3	4
4. I feel strained	1	2	3	4
5. I feel at ease	1	2	3	4
6. I feel upset	1	2	3	4
7. I am presently worrying over possible misfortunes	1	2	3	4
8. I feel satisfied	1	2	3	4
9. I feel frightened	1	2	3	4
10. I feel comfortable	1	2	3	4
11. I feel self-confident	1	2	3	4
12. I feel nervous	1	2	3	4
13. I am jittery	1	2	3	4
14. I feel indecisive	1	2	3	4
15. I am relaxed	1	2	3	4
16. I feel content	1	2	3	4
17. I am worried	1	2	3	4
18. I feel confused	1	2	3	4
19. I feel steady	1	2	3	4
20. I feel pleasant	1	2	3	4

SELF-EVALUATION QUESTIONNAIRE

STAI Form Y-2

Name____Date_________

DIRECTIONS

A number of statements which people have used to describe themselves are given below. Read each statement and then circle the appropriate number to the right of the statement to indicate how you *generally* feel. There are no right or wrong answers. Do not spend too much time on any one statement but give the answer which seems to describe how you generally feel.

	ALMOST NEVER	SOMETIMES	OFTEN	ALMOST ALWAYS
21. I feel pleasant	1	2	3	4
22. I feel nervous and restless	1	2	3	4
23. I feel satisfied with myself	1	2	3	4
24. I wish I could be as happy as others seem to be	1	2	3	4
25. I feel like a failure	1	2	3	4
26. I feel rested	1	2	3	4
27. I am "calm, cool, and collected"	1	2	3	4
28. I feel that difficulties are piling up so that I cannot overcome them	1	2	3	4
29. I worry too much over something that really doesn't matter	1	2	3	4
30. I am happy	1	2	3	4
31. I have disturbing thoughts	1	2	3	4
32. I lack self-confidence	1	2	3	4
33. I feel secure	1	2	3	4
34. I make decisions easily	1	2	3	4
35. I feel inadequate	1	2	3	4
36. I am content	1	2	3	4
37. Some unimportant thought runs through my mind and bothers me	1	2	3	4
38. I take disappointments so keenly that I can't put them out of my mind	1	2	3	4
39. I am a steady person	1	2	3	4
40. I get in a state of tension or turmoil as I think over my recent concerns and interests	1	2	3	4

State-Trait Anxiety Inventory for Adults Scoring Key (Form Y-1, Y-2)

Developed by Charles D. Spielberger in collaboration with R.L. Gorsuch, R. Lushene, P.R. Vagg, and G.A. Jacobs

To use this stencil, fold this sheet in half and line up with the appropriate test side, either Form Y-1 or Form Y-2. Simply total the scoring **weights** shown on the stencil for each response category. For example, for question # 1, if the respondent marked 3, then the **weight** would be **2**. Refer to the manual for appropriate normative data.

Form Y-1	NOT AT ALL	SOMEWHAT	MODERATELY SO	VERY MUCH SO
1.	**4**	**3**	**2**	**1**
2.	**4**	**3**	**2**	**1**
3.	**1**	**2**	**3**	**4**
4.	**1**	**2**	**3**	**4**
5.	**4**	**3**	**2**	**1**
6.	**1**	**2**	**3**	**4**
7.	**1**	**2**	**3**	**4**
8.	**4**	**3**	**2**	**1**
9.	**1**	**2**	**3**	**4**
10.	**4**	**3**	**2**	**1**
11.	**4**	**3**	**2**	**1**
12.	**1**	**2**	**3**	**4**
13.	**1**	**2**	**3**	**4**
14.	**1**	**2**	**3**	**4**
15.	**4**	**3**	**2**	**1**
16.	**4**	**3**	**2**	**1**
17.	**1**	**2**	**3**	**4**
18.	**1**	**2**	**3**	**4**
19.	**4**	**3**	**2**	**1**
20.	**4**	**3**	**2**	**1**

Form Y-2	ALMOST NEVER	SOMETIMES	OFTEN	ALMOST ALWAYS
21.	**4**	**3**	**2**	**1**
22.	**1**	**2**	**3**	**4**
23.	**4**	**3**	**2**	**1**
24.	**1**	**2**	**3**	**4**
25.	**1**	**2**	**3**	**4**
26.	**4**	**3**	**2**	**1**
27.	**4**	**3**	**2**	**1**
28.	**1**	**2**	**3**	**4**
29.	**1**	**2**	**3**	**4**
30.	**4**	**3**	**2**	**1**
31.	**1**	**2**	**3**	**4**
32.	**1**	**2**	**3**	**4**
33.	**4**	**3**	**2**	**1**
34.	**4**	**3**	**2**	**1**
35.	**1**	**2**	**3**	**4**
36.	**4**	**3**	**2**	**1**
37.	**1**	**2**	**3**	**4**
38.	**1**	**2**	**3**	**4**
39.	**4**	**3**	**2**	**1**
40.	**1**	**2**	**3**	**4**
